# Environment and Health: Not Only Cancer

**DOI:** 10.3390/ijerph13070724

**Published:** 2016-07-19

**Authors:** Annamaria Colao, Giovanna Muscogiuri, Prisco Piscitelli

**Affiliations:** 1 Department of Clinical Medicine and Surgery, University Federico II School of Medicine, Via Pansini 5, Naples 80131, Italy; 2 Southern Italy Hospital Institute (IOS), Medicina Futura Research, Naples 80143, Italy; giovanna.muscogiuri@gmail.com (G.M.); prisco.piscitelli@tiscali.it (P.P.)

**Keywords:** environment and health, pollution, cancer, cardiovascular and respiratory diseases, metabolic diseases, thyroid dysfunctions, occupational exposures and diseases

## Abstract

The Hippocratic tradition emphasized environmental causes of diseases and the need for harmony between the individual and the natural environment as the right philosophy to maintain a good health status. Public awareness and scientific attention concerning environmental pollution is usually focused on the consequent increased risk of developing cancer. Air pollution has been recognized by the World Health Organization (WHO) to cause cardiovascular and respiratroy diseases, as well as lung cancer, after acute/chronic exposure to fine particulates (PM_2.5_ and PM_10_) even at concentrations which are 50% lower than those accepted as legal limits in many developed countries. An increase of 10 µg/m^3^ of PM_2.5_ produces a +4%–6% of overall mortality, a +10% of cardiovascular disease prevalence (arithmyas, acute myocardial infarctions, and heart failure) and a +22% of lung cancer prevalence. In addition to these chronic effects, acute hospitalizations are also affected, especially among susceptible populations such as children and diabetic patients. Water and soil contamination also have an additional detrimental effect on people’s health. Other issues concerning environment contamination and human health include male/female fertility, metabolic and thyroid conditions, but also professional exposures resulting in occupational diseases. Moreover, in the perspective of “gender medicine”, different acute or chronic effects of environmental pollution should be specifically assessed both in men and in women. This special issue on “Environmental Diseases” is aimed at providing a global overview about different threats to human health possibily originating from environmental contamination.

## 1. Introduction

The 2013 European Environment Agency (EEA) “*Air quality in Europe 2015 Report*” [1] indicates that about 80% of people living in European cities are exposed to high concentrations of fine particulates (PM_2.5_ and PM_10_), known to have carcinogenic effects for humans (International Agency for Cancer Research, IARC position 2015) [2]. It is noteworthy that the 2005 World Health Organization (WHO) guidelines have recognized that PM_2.5_ and PM_10_ particulates cause negative effects on human health (in terms of cancers, respiratory, and cardiovascular diseases) even at concentrations which are 50% lower than that which is considered acceptable within the current European legal limits and environmental laws of Member States [3]. These WHO indications have been confirmed by the findings of large studies such as ESCAPE (European Study of Cohorts for Air Pollution Effects), which started in 2008 to evaluate long-term effects of air pollution on European citizens, as well as a large longitudinal study carried out on one million people living in Rome to assess the “weight” of PM_2.5_ and NO_2_ in the overall mortality registered in the last decade [4,5,6].

These studies have estimated that each increase of 10 µg/m^3^ of PM_2.5_ results in an increase of 4%–6% of the overall mortality, a 10% increase of cardiovascular disease prevalence (arithmyas, acute myocardial infarctions, and heart failure), and up to 22% increase of lung cancer prevalence, as chronic effects [4,5]. Acute effects of air pollution (both in terms of mortality and hospital admissions) have been investigated by a *MedParticles* study, which confirmed the association between PM_10_ and PM_2.5_ particulate concentrations and hospitalizations due to respiratory diseases and asthma re-acutizations in the general population, including diabetic patients and children [7].

In addition to cardiovascular or respiratory diseases and lung cancer resulting from air pollution, other issues concerning environmental contamination and human health include male/female fertility, metabolic and thyroid conditions, and also professional exposures resulting in occupational diseases. Despite all this, public awareness and scientific attention concerning environmental pollution is usually focused on the consequent increased risk of developing cancer. This special issue on “Environmental Diseases” is aimed at providing a global overview about different threats to human health originating from environmental contamination.

### 1.1. Air Pollution and Human Health: Increased Risk of Cancer, Cardiovascular and Respiratory Diseases

Reliable evidence is now available in the medical literature regarding the association between exposure to fine particulate (both acute and chronic) and human health. The “National Morbidity, Mortality, and Air Pollution Study” (NMMAPS) has assessed the negative effects of exposure to fine particulate (PM_10_ and PM_2.5_) on mortality due to cardiovascular and respiratory diseases by examining temporal series of the largest U.S. cities [8,9]. Negative effects on human health from short-term exposure to PM_2.5_ were observed by Ostro et al. in a specific assessment involving nine cities in California, showing a clear association between an increase of 15 µg/m^3^ of PM_2.5_ and mortality (+0.61% for overall causes; +0.70% for cardiovascular conditions; +2.05% for respiratory diseases) [10].

These results were confirmed in European settings by the APHEA/APHEA-2 studies (Air Pollution and Health, a European Approach, concerning the association between PM_10_ and NO_2_ short-term exposures and increases in overall mortality) [11,12], the MISA study (Italian Meta-Analysis on Short-Term Air pollution, involving 15 Italian cities with a total of 9.1 million inhabitants characterized for mortality due to cardiovascular and respiratory conditions) [13], the SISTI study (Italian Survey on Susceptibility to High Temperatures and Air Pollution, carried out in 9 cities) [14], and the EpiAir Project (a survey matching environmental and healthcare datasets concerning 300,000 people aged <35 years old, living in 10 Italian cities in the years 2001–2005) [15]. The acute exposure to hotspots of air pollutants such as fine particulates (PM_10_), NO_2_, and O_3_ has been shown to increase hospital admissions for cardiac diseases and respiratory complaints, especially in sensitive subgroups (i.e., asthmatic patients) [16,17,18].

In a German study, women aged 50–59 years old living within 50 m from heavily trafficked roads (in the hypothesis of a long-term exposure) had a 70% higher probability of mortality as a consequence of cardiovascular or respiratory diseases than those living far away from urban traffic (odds ratio OR: 1.70; 95%CI 1.02–2.81) [19]. In a systematic review of European and American studies, the risk of developing cancer or cardiovascular/respiratory diseases following long term exposures to air pollution was found to be 6% higher for each increase of 10 μg/m^3^ in the atmospheric concentration of PM_2.5_, with this result being independent of age, gender, and geographic area [20]. Similarly, a 16-year follow up carried out on 500,000 people living in U.S. urban areas has shown—per each increase of 10 μg/m^3^ of PM_2.5_—an increase of 6%, 9%, and 14% in the overall mortality (all causes), lung cancer prevalence, and cardiopulmonary conditions, respectively [21].

A Dutch survey on 120,852 people showed similar increased risks concerning long term exposures to NO_2_ (+5% in overall mortality and cardiopulmonary diseases, but a +20% increased risk of mortality due to of lung cancer after chronic exposure to air pollution originating from urban traffic) [22,23]. A recent 18-year follow-up carried out on German women has pointed out significant increases in the risk of mortality due to cardiopulmonary diseases and lung cancer as a consequence of minimally increased levels of PM_10_ atmospheric concentrations (7 μg/m^3^) [24]. A specific association between PM_2.5_ or NO_2_ and an increased risk of mortality from cardiovascular diseases and pulmonary cancer has been highlighted also in a study carried out in Rome and another study involving 20 US cities [5,25]. Living in urban areas has been proven to be more frequently associated with the presence of mutagenic markers indicating an early biologic effect (*sister chromatide exchange*) or with asthma/respiratory conditions than living in sub-urban or rural areas [26,27]. Vineis et al. (European Prospective Investigation on Cancer and Nutrition, EPIC) have observed a significant association between chronic exposures to NO_2_ (concentrations > 30 μg/m^3^) and lung cancer (OR: 1.30; 95%CI 1.02–1.66) [28].

The same EPIC population has allowed sub-analyses that have confirmed the higher risk of traffic-exposed workers developing lung cancer [29]. Concerning professional exposures, a study of a cohort of 230,000 bus drivers showed that a significant increase of mortality due to cancer is present in workers exposed to traffic for 30 or more years [30]. Another population characterized by higher susceptibility—other than workers—is that of children, whom have been shown to suffer more than adults from asthma and other respiratory conditions when exposed to high concentrations of fine particulate and NO_2_ (with these pollutants also being associated with increases in overall pediatric mortality rates) [31,32,33,34,35,36,37,38,39,40,41,42,43,44].

### 1.2. Effects on Human Health of Soil, Water, and Food Chain Contamination

The issue of potential effects of illegal toxic waste dumping has been addressed in several studies [45,46,47,48,49,50,51]. Increased cancer mortality rates (lung, liver, gastric, kidney, and bladder cancer) and clusters of malformations (at limb, cardiovascular, and urogenital systems) have been documented in some areas characterized by the widespread illegal practice of dumping toxic and urban waste [47]. While many illegal waste sites have been found to contain dangerous chemicals such as mineral oil, lead, mercury, aluminum residuals, arsenic, and tire residuals, even the presence of authorized urban waste dumping sites has been recently proposed for association with a higher risk of gastric cancer [52]. The scientific community is aware that persistent pollutants can contaminate the food chain leading to a bioaccumulation phenomenon in animals [53] and even in humans [54,55,56,57,58,59,60,61,62,63,64,65]. The presence of dioxin and polychlorobiphenyles PCBs have been documented in breast milk of young women in many industrialized countries, and this has been proposed as a potential reliable model for the assessment of people’s exposure to environmental contaminants. A systematic review on 50 studies of waste landfills and incinerators has found a relationship between the risk of congenital anomalies in people living in the proximity of special waste landfills [66]. Heavy industrial contamination resulting in the spread of arsenic, lead, mercury, nickel, cadmium, polycyclic aromatic hydrocarbons PAHs, and halogenated compounds has been indicated by many authors to be responsible for the excesses of mortality and morbidity of resident populations [67,68,69,70,71,72,73,74,75,76,77,78,79,80,81,82]. Evidence is also available concerning water and soil contamination due to the massive use of pesticides in intensive agriculture [83].

### 1.3. Effects of Endocrine Disrupting Chemicals on Cardiovascular System and Metabolism

Recent evidence supports a role of phtalates in the pathogenesis of atherosclerosis and hypertension. It is well known that phtalates are commonly found in several household products such as food packaging, furniture, and toys. Humans are exposed to phtalates through different means such as inhalation, ingestion, and dermal exposure [84]. Because of the abundance of plastic in our society, this exposure to phtalates is ubiquitous. The PIVUS study (Prospective Investigation of the Vasculature in Uppsala) demonstrated a significant inverse correlation between mono-ethylphthalate and both systolic blood pressure (SBP) and diastolic blood pressure (DBP) increases [85]. Phtalate metabolites were associated to SBP but none of these metabolites was related to DBP in the study by Trasande et al. [86]. A strong relationship between di-2-ethylhexyl phthalate and blood pressure was found in a subsample of US children aged 6–19 years who participated in the National Health and Nutrition Examination Survey between 2003 and 2008. For each log unit increase in di-2-ethylhexyl phthalate metabolites, a 0.041 SD unit increase in systolic BP z-score was identified [86]. Monobenzylphthalate has been associated with increased DBP and increased risk of pregnancy-induced hypertensive diseases in 369 women of the Health Outcomes and Measures of the Environment study, a prospective birth cohort of low-risk pregnant women recruited between March 2003 and January 2006 [87]. In the study performed by Lind et al., mono-methyl phthalate was related to carotid plaques in an inverted U-shaped manner while mono-isobutyl phthalate and mono-methyl phthalate levels were associated to the echogenicity of the plaque [88]. The phtalate-related hypertension could be related to the increased plaque echogenicity and intima-media thickening and echogenicity that are more likely to happen in subjects exposed to phtalates [88].

By analyzing 2003–2004 National Health and Nutrition Examination Survey NHANES data, Lang et al. found that one standard deviation increase in urinary Bisphenol A concentration was associated with an increase in cardiovascular diseases such as coronary heart disease, myocardial infarction, and angina (OR 1.39, 95%CI 1.18–1.63) [89]. If Bisphenol A exposure is indeed associated with cardiovascular diseases, it would be a major public health problem since Bisphenol A exposure is ubiquitous. In fact, Bisphenol A-based polycarbonate plastics are used in plastic bottles, food containers, and optical disks, while epoxy resin-containing Bisphenol A is used in water pipe lining [90]. Bisphenol A has been associated with insulin resistance and it has also been suspected to modulate adiponectin and resistin gene expression in obese children, thus being involved in the pathophysiology of type 2 diabetes [91,92].

## 2. Conclusions

Developed countries and emerging economies need to take heed of the mounting evidence against pollution because even small reductions of environmental pollutants could confer substantial population health benefits. Moreover, in the perspective of “gender medicine”, different acute or chronic effects of environmental pollution should be specifically assessed both in men and in women as well as in children and other specifically susceptible populations.

## References

[1] European Environmental Agency Air Quality in Europe 2015 Report. http://www.eea.europa.eu//publications/air-quality-in-europe-2015#tab-data-references.

[2] International Agency for Research on Cancer (2014). IARC Monographs on the Evaluation of Carcinogenic Risks to Humans.

[3] World Health Organization (2006). Air Quality Guidelines—Global Update 2005. http://whqlibdoc.who.int/hq/2006/WHO_SDE_PHE_OEH_06.02_eng.pdf.

[4] Raaschou-Nielsen O., Andersen Z.J., Beelen R., Samoli E., Stafoggia M., Weinmayr G., Hoffmann B., Fischer P., Nieuwenhuijsen M.J., Brunekreef B. (2013). Air pollution and lung cancer incidence in 17 European cohorts: Prospective analyses from the European Study of Cohorts for Air Pollution Effects (ESCAPE). Lancet Oncol..

[5] Cesaroni G., Badaloni C., Gariazzo C., Stafoggia M., Sozzi R., Davoli M., Forastiere F. (2013). Long-term exposure to urban air pollution and mortality in a cohort of more than a million adults in Rome. Environ. Health Perspect..

[6] World Health Organization (2013). Review of Evidence on Health Aspects of Air Pollution—REVIHAAP. http://www.euro.who.int/_data/assets/pdf_file/0020/182432/e96762-final.pdf.

[7] Samoli E., Stafoggia M., Rodopoulou S., Ostro B., Declercq C., Alessandrini E., Díaz J., Karanasiou A., Kelessis A.G., le Tertre A. (2013). Associations between fine and coarse particles and mortality in Mediterranean cities: Results from the MED-PARTICLES project. Environ. Health Perspect..

[8] Dominici F., McDermott A., Daniels M., Zeger S.L., Samet J.M. (2005). Revised analyses of the national morbidity, mortality, and air pollution study: Mortality among residents of 90 cities. J. Toxicol. Environ. Health A.

[9] Dominici F., Peng R.D., Bell M.L., Pham L., McDermott A., Zeger S.L., Samet J.M. (2006). Fine particulate air pollution and hospital admission for cardiovascular and respiratory diseases. JAMA.

[10] Ostro B., Feng W.-Y., Broadwin R., Lipsett M. (2007). The effects of components of fine particulate air pollution on mortality in California: Results from CALFINE. Environ. Health Perspect..

[11] Gryparis A., Forsberg B., Katsouyanni K., Analitis A., Touloumi G., Schwartz J., Samoli E., Medina S., Anderson H.R., Niciu E.M. (2004). Acute effects of ozone on mortality from the “Air pollution and health: A European approach” project. Am. J. Respir. Crit. Care Med..

[12] Anderson H.R., Spix C., Medina S., Schouten J.P., Castellsague J., Rossi G., Zmirou D., Touloumi G., Wojtyniak B., Ponka A. (1997). Air pollution and daily admissions for chronic obstructive pulmonary disease in 6 European cities: Results from the APHEA project. Eur. Respir. J..

[13] Biggeri A., Bellini P., Terracini B. (2004). Meta-analysis of the Italian studies on short term effects of air pollution—MISA 1996–2002. Epidemiol. Prev..

[14] Forastiere F., Stafoggia M., Berti G., Bisanti L., Cernigliaro A., Chiusolo M., Mallone S., Miglio R., Pandolfi P., Rognoni M. (2008). Particulate matter and daily mortality: A case-crossover analysis of individual effect modifiers. Epidemiology.

[15] Faustini A., Stafoggia M., Berti G., Bisanti L., Chiusolo M., Cernigliaro A., Mallone S., Primerano R., Scarnato C., Simonato L. (2011). The relationship between ambient particulate matter and respiratory mortality: A multi-city study in Italy. Eur. Respir. J..

[16] Vigotti M.A., Chiaverinib F., Biagiolac P., Rossic G. (2007). Urban air pollution and emergency visits for respiratory complaints in Pisa, Italy. J. Toxicol. Environ. Health.

[17] Colais P., Faustini A., Stafoggia M., Berti G., Bisanti L., Cadum E., Cernigliaro A., Mallone S., Pacelli B., Serinelli M. (2012). Particulate air pollution and hospital admissions for cardiac diseases in potentially sensitive subgroups. Epidemiology.

[18] Colais P., Serinelli M., Faustini A., Stafoggia M., Randi G., Tessari R., Chiusolo M., Pacelli B., Mallone S., Vigotti M.A. (2009). Air pollution and urgent hospital admissions in nine Italian cities. Results of the EpiAir Project. Epidemiol. Prev..

[19] Gehring U., Heinrich J., Krämer U., Grote V., Hochadel M., Sugiri D., Kraft M., Rauchfuss K., Eberwein H.G., Wichmann H.-E. (2006). Long-term exposure to ambient air pollution and cardiopulmonary mortality in women. Epidemiology.

[20] Chen H. (2008). A systematic review of the relation between long-term exposure to ambient air pollution andchronic diseases. Rev. Environ. Health.

[21] Pope C.A., Burnett R.T., Thun M.J., Calle E.E., Krewski D., Ito K., Thurston G.D. (2002). Lung cancer, cardiopulmonary mortality, and long-term exposure to fine particulate air pollution. JAMA.

[22] Brunekreef B., Beelen R., Hoek G., Schouten L., Bausch-Goldbohm S., Fischer P., Armstrong B., Hughes E., Jerrett M., van den Brandt P. (2009). Effects of long-term exposure to traffic-related air pollution on respiratory and cardiovascular mortality in The Netherlands: The NLCS-AIR study. Res. Rep. Health Eff. Inst..

[23] Beelen R., Hoek G., van den Brandt P.A., Goldbohm R.A., Fischer P., Schouten L.J., Jerrett M., Hughes E., Armstrong B., Brunekreef B. (2008). Long-term effects of traffic-related air pollution on mortality in a Dutch cohort (NLCSAIR study). Environ. Health Perspect..

[24] Heinrich J., Thiering E., Rzehak P., Kramer U., Hochadel M., Rauchfuss K.M., Gehring U., Wichmann H.-E. (2013). Long-term exposure to NO_2_ and PM_10_ and all-cause and cause-specific mortality in a prospective cohort of women. Occup. Environ. Med..

[25] Samet J.M., Dominici F., Curriero F.C., Coursac I., Zeger S.L. (2000). Fine particulate, air pollution and mortality in 20 U.S. cities, 1987–1994. N. Engl. J. Med..

[26] Barale R., Chelotti L., Davini T., Landi S. (1998). Sister chromatid exchange and micronucleus frequency in human lymphocytes of 1650 subjects in an Italian population: II. Contribution of sex, age, and lifestyle. Environ. Mol. Mutagen..

[27] Iversen L., Hannaford P.C., Price D., Godden D.J. (2005). Is living in a rural area good for your respiratory health? Results from a cross-sectional study in Scotland. Chest.

[28] Vineis P., Hoek G., Krzyzanowski M., Vigna-Taglianti F., Veglia F., Airoldi L., Autrup H., Dunning A., Garte S., Hainaut P. (2006). Air pollution and risk of lung cancer in a prospective study in Europe. Int. J. Cancer.

[29] Palli D., Saieva C., Munnia A., Peluso M., Grechi D., Zanna I., Caini S., Decarli A., Sera F., Masal G. (2008). DNA adducts and PM(10) exposure in traffic-exposed workers and urban residents from the EPIC-Florence City study. Sci. Total Environ..

[30] Merlo D.F., Stagi E., Fontana V., Consonni D., Gozza C., Garrone E., Bertazzi P.A., Pesatori A.C. (2010). A historical mortality study among bus drivers and bus maintenance workers exposed to urban air pollutants in the city of Genoa, Italy. Occup. Environ. Med..

[31] Scheers H., Mwalili S.M., Faes C., Fierens F., Nemery B., Nawrot T.S. (2011). Does air pollution trigger infant mortality in Western Europe? A Case-crossover study. Environ. Health Perspect..

[32] Valent F., Little D., Bertollini R., Nemer L.E., Barbone F., Tamburlini G. (2004). Burden of disease attributable to selected environmental factors and injury among children and adolescents in Europe. Lancet.

[33] De Medeiros A.P., Gouveia N., Machado R.P.P., de Souza M.R., Alencar G.P., Novaes H.M.D., de Almeida M.F. (2009). Traffic-related air pollution and perinatal mortality: A case-control study. Environ. Health Perspect..

[34] Romeo E., De Sario M., Forastiere F., Compagnucci P., Stafoggia M., Bergamaschi A., Perucci C.A. (2006). PM_10_ exposure and asthma exacerbations in pediatric age: A meta-analysis of panel and time-series studies. Epidemiol. Prev..

[35] Linares C., Díaz J. (2009). Impact of particulate matter with diameter of less than 2.5 microns (PM_2.5_) on daily hospital admissions in 0-10-year-olds in Madrid, Spain (2003–2005). Gac. Sanit..

[36] Andersen Z.J., Wahlin P., Raaschou-Nielsen O., Loft S. (2008). Size distribution and total number concentration of ultrafine and accumulation mode particles and hospital admissions in children and the elderly in Copenhagen, Denmark. Occup. Environ. Med..

[37] Annesi-Maesano I., Moreau D., Caillaud D., Lavaud F., Le Moullec Y., Taytard A., Pauli G., Charpin D. (2007). Residential proximity fine particles related to allergic sensitisation and asthma in primary school children. Respir. Med..

[38] Pénard-Morand C., Raherison C., Charpin D., Kopferschmitt C., Lavaud F., Caillaud D., Annesi-Maesano I. (2010). Long-term exposure to close-proximity air pollution and asthma and allergies in urban children. Eur. Respir. J..

[39] Brauer M., Hoek G., Smit H.A., de Jongste J.C., Gerritsen J., Postma D.S., Kerkhof M., Brunekreef B. (2007). Air pollution and development of asthma, allergy and infections in a birth cohort. Eur. Respir. J..

[40] Ciccone G., Forastiere F., Agabiti N., Biggeri A., Bisanti L., Chellini E., Corbo G., Dell’Orco V., Dalmasso P., Volante T.F. (1998). Road traffic and adverse respiratory effects in children. SIDRIA Collaborative Group. Occup. Environ. Med..

[41] Cibella F., Cuttitta G., La Grutta S., Melis M.R., Lospalluti M.L., Uasuf C.G., Bucchieri S., Viegi G. (2011). Proportional Venn diagram and determinants of allergic respiratory diseases in Italian adolescents. Pediatr. Allergy Immunol..

[42] Gauderman W.J., Vora H., McConnell R., Berhane K., Gilliland F., Thomas D., Lurmann F., Avol E., Kunzli N., Jerrett M. (2007). Effect of exposure to traffic on lung development from 10 to 18 years of age: A cohort study. Lancet.

[43] Pope C.A. (2000). Epidemiology of fine particulate air pollution and human health: Biologic mechanism and who’s at risk?. Environ. Health Perspect..

[44] Chuang K.J., Chan C.C., Su T.C., Lee C.T., Tang C.S. (2007). The effect of urban air pollution on inflammation, oxidative stress, coagulation and autonomic dysfunction in young adults. Am. J. Respir. Crit. Care Med..

[45] Martuzzi M., Mitis F., Biggeri A., Terracini B., Bertollini R. (2002). Environment and health status of the population in areas with high risk of environmental crisis in Italy. Epidemiol. Prev..

[46] Martuzzi M., Mitis F., Bianchi F., Comba P., Fazzo L. (2009). Cancer mortality and congenital anomalies in a region of Italy with an intense pressure due to waste. Occup. Environ. Med..

[47] Fazzo L., Belli S., Minichilli F., Mitis F., Santoro M., Martina L., Pizzuti R., Comba P., Martuzzi M., Bianchi F. (2008). Cluster analysis of mortality and malformations in the provinces of Naples and Caserta (Campania Region). Ann. 1st Super Sanità.

[48] Mazza A., Piscitelli P., Neglia C., della Rosa G., Iannuzzi L. (2015). Illegal Dumping of Toxic Waste and Its Effect on Human Health in Campania, Italy. Int. J. Environ. Res. Public Health.

[49] Altavista P., Belli S., Bianchi F., Pizzuti R. (2004). Cause of specific mortality in a district of Campania Region with high number of waste dump sites. Epidemiol. Prev..

[50] Comba P., Bianchi F., Fazzo L., Martina L., Menegozzo M., Minichilli F., Mitis F., Musmeci L., Pizzuti R., Santoro M., Trinca S. (2006). Cancer mortality in an area of Campania (Italy) characterized by multiple toxic dumping sites. Ann. N. Y. Acad. Sci..

[51] Pirastu R., Pasetto R., Zona A., Ancona C., Iavarone I., Martuzzi M., Comba P. (2013). The health profile of populations living in contaminated sites: SENTIERI approach. J. Environ. Public Health.

[52] Di Ciaula A. (2016). Increased deaths from gastric cancer in communities living close to waste landfills. Int. J. Environ. Health Res..

[53] Esposito M., Cavallo S., Serpe F.P., D’Ambrosio R., Gallo P., Colarusso G., Pellicanò R., Baldi L., Guarino A., Serpe L. (2009). Levels and congener profiles of polychlorinated dibenzo-p-dioxins, polychlorinated dibenzofurans and dioxin-like polychlorinated biphenyls in cow’s milk collected in Campania, Italy. Chemosphere.

[54] Rivezzi G., Piscitelli P., Scortichini G., Giovannini A., Diletti G., Migliorati G., Ceci R., Rivezzi G., Cirasino L., Carideo P. (2013). A general model of dioxin contamination in breast milk: Results from a study on 94 women from the Caserta and Naples areas in Italy. Int. J. Environ. Res. Public Health.

[55] Ulaszewska M., Zuccato E., Capri E., Iovine R., Colombo A., Rotella G., Generoso C., Grassi P., Melis M., Fanelli R. (2011). The effect of waste combustion on the occurrence of polychlorinated dibenzo-p-dioxins (PCDDs), polychlorinated dibenzofurans (PCDFs) and polychlorinated biphenyls (PCBs) in breast milk in Italy. Chemosphere.

[56] Giovannini A., Rivezzi G., Carideo P., Ceci R., Diletti G., Ippoliti C., Migliorati G., Piscitelli P., Ripani A., Salini R. (2014). Dioxins levels in breast milk of women living in Caserta and Naples: Assessment of environmental risk factors. Chemosphere.

[57] Lignell S., Aune M., Darnerud P.O., Cnattingius S., Glynn A. (2009). Persistent organochlorine and organobromine compounds in mother’s milk from Sweden 1996–2006: Compound-specific temporal trends. Environ. Res..

[58] Li J., Zhang L., Wu Y., Liu Y., Zhou P., Wen S., Liu J., Zhao Y., Li X. (2009). A national survey of polychlorinated dioxins, furans (PCDD/Fs) and dioxin-like polychlorinated biphenyls (dl-PCBs) in human milk in China. Chemosphere.

[59] Becher G., Skaare J.U., Polder A., Slettene B., Rossland O.J., Hansen H.K., Ptashekas J. (1995). PCDDs, PCDFs, and PCBs in human milk from different parts of Norway and Lithuania. J. Toxicol. Environ. Health.

[60] Costopoulou D., Vassiliadou I., Papadopoulos A., Makropoulos V., Leondiadis L. (2006). Levels of dioxins, furans and PCBs in human serum and milk of people living in Greece. Chemosphere.

[61] Raab U., Schwegler U., Preiss U., Albrecht M., Fromm H. (2007). Bavarian breast milk survey—Pilot study and future developments. Int. J. Hyg. Environ. Health.

[62] Harden F.A., Toms L.M., Symons R., Fürst P., Berry Y., Müller J.F. (2007). Evaluation of dioxin-like chemicals in pooled human milk samples collected in Australia. Chemosphere.

[63] Wittsiepe J., Fürst P., Schrey P., Lemm F., Kraft M., Eberwein G., Winneke G., Wilhelm M. (2007). PCDD/F and dioxin-like PCB in human blood and milk from German mothers. Chemosphere.

[64] Kedem M.H., Mandel D., Domani K.A., Mimouni F.B., Shay V., Marom R., Dollberg S., Herman L., Lubetzky R. (2013). The effect of advanced maternal age upon human milk fat content. Breastfeed. Med..

[65] Bianco G., Zianni R., Anzillotta G., Palma A., Vitacco V., Scrano L., Cataldi T.R. (2013). Dibenzo-p-dioxins and dibenzofurans in human breast milk collected in the area of Taranto (Southern Italy): First case study. Anal. Bioanal. Chem..

[66] Ashworth D.C. (2014). Waste incineration and adverse birth and neonatal outcomes: A systematic review. Environ. Int..

[67] Cousins I.T., Vestergren R., Wang Z., Scheringer M., McLachlan M.S. (2016). The precautionary principle and chemicals management: The example of perfluoroalkyl acids in groundwater. Environ. Int..

[68] Pulkrabov J., Stupak M., Svarcov A., Rossner P., Rossnerov A., Ambroz A., Sram R., Hajslov J. (2016). Relationship between atmospheric pollution in the residential area and concentrations of polycyclic aromatic hydrocarbons (PAHs) in human breast milk. Sci. Total Environ..

[69] Park J.D., Choi S.J., Choi B.S., Lee C.R., Kim H., Kim Y.D., Park K.S., Lee Y.J., Kang S., Lim K.M. (2016). Arsenic levels in the groundwater of Korea and the urinary excretion among contaminated area. J. Expo. Sci. Environ. Epidemiol..

[70] Liu F.F., Wang J.P., Zheng Y.J., Ng J.C. (2013). Biomarkers for the evaluation of population health status 16 years after the intervention of arsenic-contaminated groundwater in Xinjiang, China. J. Hazard Mater..

[71] O’Reilly J., Watts M.J., Shaw R.A., Marcilla A.L., Ward N.I. (2010). Arsenic contamination of natural waters in San Juan and La Pampa, Argentina. Environ. Geochem. Health.

[72] Chakraborti D., Rahman M.M., Das B., Murrill M., Dey S., Mukherje S.C., Dhar R.K., Biswas B.K., Chowdhury U.K., Roy S. (2010). Status of groundwater arsenic contamination in Bangladesh: A 14-year study report. Water Res..

[73] Cho Y., Seo S., Choi S.H., Lee S., Kim K., Kim H.J., Choi J.W. (2013). Association of arsenic levels in soil and water with urinary arsenic concentration of residents in the vicinity of closed metal mines. Int. J. Hyg. Environ. Health.

[74] Mohankumar K., Hariharan V., Prasada Rao N. (2016). Heavy Metal Contamination in Groundwater around Industrial Estate vs. Residential Areas in Coimbatore, India. J. Clin. Diagn. Res..

[75] Steindor K.A., Franiel I.J., Bierza W.M., Pawlak B., Palowski B.F. (2016). Assessment of heavy metal pollution in surface soils and plant material in the post-industrial city of Katowice, Poland. J. Environ. Sci. Health A Toxic Hazard Subst. Environ. Eng..

[76] Liu H., Probst A., Liao B. (2005). Metal contamination of soils and crops affected by the Chenzhou lead/zinc mine spill (Hunan, China). Sci. Total Environ..

[77] Hu X., Ding Z. (2009). Lead/cadmium contamination and lead isotopic ratios in vegetables grown in peri-urban and mining/smelting contaminated sites in Nanjing, China. Bull. Environ. Contam. Toxicol..

[78] Anju M., Banerjee D.K. (2011). Associations of cadmium, zinc, and lead in soils from a lead and zinc mining area as studied by single and sequential extractions. Environ. Monit. Assess..

[79] Di Leonardo R., Mazzola A., Tramati C.D., Vaccaro A., Vizzini S. (2014). Highly contaminated areas as sources of pollution for adjoining ecosystems: The case of Augusta Bay (Central Mediterranean). Mar. Pollut. Bull..

[80] Carrer S., Leardi R. (2006). Characterizing the pollution produced by an industrial area: Chemometric methods applied to the Lagoon of Venice. Sci. Total Environ..

[81] Gibicar D. (2009). Human exposure to mercury in the vicinity of chlor-alkalin plant. Environ. Res..

[82] Fano V., Forastiere F., Papini P., Tancioni V., Di Napoli A., Perucci C.A. (2006). Mortality and hospital admissions in the industrial area of Civitavecchia, 1997–2004. Epidemiol. Prev..

[83] Forastiere F. (2015). Pesticides, food and health. Epidemiol. Prev..

[84] Wams T.J. (1987). Diethylhexylphthalate as an environmental contaminant: A review. Sci. Total Environ..

[85] Olsén L., Lind L., Lind P.M. (2012). Associations between circulating levels of bisphenol A and phthalate metabolites and coronary risk in the elderly. Ecotoxicol. Environ. Saf..

[86] Trasande L., Sathyanarayana S., Spanier A.J., Trachtman H., Attina T.M. (2013). Urinary phthalates are associated with higher blood pressure in childhood. J. Pediatr..

[87] Werner E.F. (2015). The association between maternal urinary phthalate concentrations and blood pressure in pregnancy: The HOME Study. Environ. Health.

[88] Lind P.M., Lind L. (2011). Circulating levels of Bisphenol A and phthalates are related to carotid atherosclerosis in the elderly. Atherosclerosis.

[89] Lang I.A., Galloway T.S., Scarlett A., Henley W.E., Depledge M., Wallace R.B., Melzer D. (2008). Association of urinary bisphenol A concentration with medical disorders and laboratory abnormalities in adults. JAMA.

[90] Burridge E. (2003). Product profile: Bisphenol A. Eur. Chem. News.

[91] Menale C., Grandone A., Nicolucci C., Cirillo G., Crispi S., di Sessa A., Marzuillo P., Rossi S., Mita D.G., Perrone L. (2016). Bisphenol A is associated with insulin resistance and modulates adiponectin and resistin gene expression in obese children. Pediatr. Obes..

[92] Chevalier N., Fénichel P. (2015). Endocrine disruptors: New players in the pathophysiology of type 2 diabetes?. Diabetes Metab..

